# Dairy intake and long-term body weight status in German children and adolescents: results from the DONALD study

**DOI:** 10.1007/s00394-021-02715-9

**Published:** 2021-10-31

**Authors:** Eva Hohoff, Ines Perrar, Nicole Jankovic, Ute Alexy

**Affiliations:** Institute of Nutritional and Food Sciences-Nutritional Epidemiology, University of Bonn, DONALD Study, Heinstück 11, 44225 Dortmund, Germany

**Keywords:** Dairy, Dairy types, Body composition, Body mass index, Children, Adolescents

## Abstract

**Purpose:**

To analyse the association between intake of total dairy (TD) and types of dairy [liquid dairy (LD), solid dairy (SD), low-fat dairy (LFD), high-fat dairy (HFD), high sugar dairy (HSD), low-sugar dairy (LSD), not fermented dairy (NFD), as well as fermented dairy (FD)] and long-term changes in body weight status and composition among children and adolescents in Germany.

**Methods:**

In total, 9999 3-day dietary records collected between 1985 and 2019 by 1126 participants (3.5–18.5 years; boys: 50.8%) of the Dortmund Nutritional and Anthropometric Longitudinally Designed (DONALD) study were analysed. Polynomial mixed-effects regression models were used to examine whether changes (median follow-up: 9 years) in the intake of TD and dairy types (in 100 g/1000 kcal total energy intake) were associated with changes in body-mass-index-standard-deviation-score (BMI-SDS); fat mass index (FMI); fat-free mass index (FFMI) over time.

**Results:**

An individual increase in TD intake was slightly but significantly associated with an increase in BMI-SDS (*β *= 0.0092; *p* = 0.0371), FMI (*β *= 0.022; *p* = 0.0162), and FFMI (*β *= 0.0156; *p* = 0.0417) after adjustment for potential confounder. Analyses for LD (BMI-SDS: *β *= 0.0139; *p* = 0.0052; FMI: *β *= 0.0258; *p* = 0.0125; FFMI: *β *= 0.0239; *p* = 0.0052) and LSD intake (BMI-SDS: *β *= 0.0132; *p* = 0.0041, FMI: *β *= 0.02; *p* = 0.0316, FFMI: *β *= 0.0183; *p* = 0.0189) showed similar results to TD. Both processing method and fat content showed no association with body composition in our analyses.

**Conclusion:**

Increases in TD, LD, and LSD intake showed small but significant increases in BMI and concomitant increases in fat mass and lean mass. However, the observed changes were too small to expect biological or physiological meaningful effects. Overall, our results showed that policies to promote dairy intake in childhood are to be welcomed, as no negative effects on body composition are expected, while the intake of important nutrients for growth is ensured. The type of dairy does not seem to matter.

**Supplementary Information:**

The online version contains supplementary material available at 10.1007/s00394-021-02715-9.

## Introduction

The high prevalence of overweight and obesity in childhood and adolescence represents a global health problem. According to the WHO reference values [1], 26.3% of children and adolescents (5–17 years) in Germany are overweight. Of those, even 8.8% are obese [2]. To counteract this development, the European Union has been providing member states with 150 million euros annually for school fruit and vegetables and 100 million euros for the distribution of school milk since 2017 with the ‘EU school schemes’. Germany alone receives around 10.4 million euros for the distribution of milk and certain dairy types in schools and kindergartens [3]. Dairy, which has long been recommended for children and adolescents because of its beneficial nutritional composition (e.g., protein or calcium content), is controversially discussed as a potential protective or risk-increasing factor for the development of overweight. In fact, some meta-analyses and systematic reviews led to some different results [4–9].

One reason for these inconsistent results could be the heterogeneity of dairy in terms of nutrient content (fat, sugar), processing methods (fermentation), and consistency (liquid, solid) [10].

Regarding the fat content, a systematic review [11] concluded that full-fat dairy products were not associated with increased weight gain or obesity in children A meta-analysis [12] in adults also could not find significant results between the intake of full-fat dairy products and low-fat dairy products on changes in body weight per serving. However, there was an inverse association between the changes in body weight for each serving of yoghurt, while the increase in each serving of cheese was positively associated.

Due to the contradictive results observed so far in the literature, the individual types of dairy are moving more into the focus of scientific interest. To clarify the role of dairy and dairy types on health requires the investigation regarding the associations between this food group on body weight status and composition [13, 14].

Due to the lack of knowledge as well as a limited number of studies during growth from childhood to adolescence, the aim of the present analyses was to investigate the prospective association between intake of total dairy and dairy types with changes in body composition among German children and adolescents. Results derived in the context of this paper are intended to contribute to the evaluation and further development of current health policies.

## Methods

### Study sample

The DONALD study is an ongoing, open cohort study started in 1985 in Dortmund, Germany. The study investigates relationships between nutrition, metabolism, growth, and development in healthy infants, children, and adolescents aged 3 months to young adulthood. Approximately 40 infants are enrolled in the study each year.

The annual examinations include 3-day weighed dietary records, anthropometric measurements, 24-h urine samples, lifestyle interviews, and medical examinations. In addition, anthropometric data as well as socio-economic and lifestyle factors of the parents were collected every 4 years. Further details are described elsewhere [15].

The ethic committee of the University of Bonn approved the study according to the guidelines of the Declaration of Helsinki. All examinations were carried out with written consent of the participants or their parents.

The study sample for the present data analysis includes all available complete dietary records of 3 days among children and adolescents (3.5–18.5 years) between 1985 and 2019 (*N* = 9999 records, *n* = 1126 participants, 51% boys).

### Dietary assessment

Dietary intakes are assessed using 3-day weighed dietary records. All food and beverages consumed on 3 consecutive days are weighed and recorded to the nearest 1 g by parents or later by the participants themselves using digital scales. If accurate weighing is not possible, semi-quantitative recording such as the number of teaspoons or glasses can be provided instead.

Energy and nutrient intake are calculated using our continuously updated in-house nutrient database LEBTAB [16]. The composition of basic foods (e.g., milk, yoghurt) is based on the German food composition tables BLS 3.02. Energy and nutrient contents of commercial food products, i.e., commercial sweetened yoghurt, milk-based desserts, or beverages, are estimated by recipe simulation using labelled ingredients and nutrients [16].

### Dairy intake

For the current analyses, total dairy (TD) was categorized into the following partly overlapping dairy types.

With regard to the way of intake:

- liquid dairy (LD) and solid dairy (SD).

With regard to the nutrient content:

- low fat dairy (LFD) and high fat dairy (HFD)
- high sugar dairy (HSD) and low sugar dairy (LSD).

With regard to the processing method:

- not fermented dairy (NFD) and fermented dairy (FD).

Detailed descriptions of total dairy and the different dairy types are given in Table 1. The daily intake of dairy and dairy types was calculated from the individual mean of the 3 day dietary records and standardized for g/1000 kcal of total energy intake (TEI).

**Table 1:** Classification of dairy products, DONALD study

	Included dairy products
Total dairy (TD)^a^	All dairy products (including dairy from cows and other mammals, such as goats or sheep)
Types of dairy	
Liquid dairy (LD)	Fresh milk, not fermented and fermented drinks (e.g., cacao, buttermilk, whey), liquid sour milk products (incl. squeeze sour milk), yoghurt drink
Solid dairy (SD)	Not fermented and fermented dairy food (e.g., yoghurt, cheese), milk for cereals or pudding
Fermented dairy (FD)	Fermented liquid and solid dairy: fermented dairy drinks (buttermilk, whey), liquid sour milk products (incl. squeeze sour milk), yoghurt drink, yoghurt, firm sour milk products, fermented desserts, fresh cheese, quark, cream fraiche, cheese (soft cheese, sliced cheese, hard cheese, processed cheese)
Not fermented dairy (NFD)	Not fermented liquid and solid dairy: milk, not fermented dairy drinks (e.g., cocoa, milk shakes), not fermented milk desserts
Low-fat dairy (LFD)^b^	Non-fermented and fermented beverage dairy, non-fermented solid dairy, fermented solid dairy (fresh cheese, quark) < 2% fat, fresh cheese, quark (< 9% fat), soft cheese, processed cheese (< 15% fat), semi-hard and hard cheese (< 18% fat)
High-fat dairy (HFD)^b^	Non-fermented and fermented beverage dairy, non-fermented solid dairy, fermented solid dairy (fresh cheese, quark) > 2% fat, fresh cheese, quark (> 9% fat), soft cheese, processed cheese (> 15% fat), semi-hard and hard cheese (> 18% fat)
Low-sugar dairy (LSD)^c,d^	Natural sugar content and added sugar < 7 g/100 g industrially sweetened dairy
High-sugar dairy (HSD)^c,d^	Added sugar > 7 g/100 g industrially sweetened dairy

Dairy products can occur in different groups

^a^Excluding cream cakes and ice cream, because they are consumed as sweets rather than to meet dairy requirements, and excluding butter

^b^Classification based on https://www.lebensmittellexikon.de/f0000170.php

^c^Including instant powders for milk (i.e., cocoa)

^d^The cut-off was set based on the first quartile (6.9 g added sugar/100 g) from all sweetened dairy products (*n* = 965) reported by the study sample

### Anthropometric measurements

The anthropometric measurements (height, weight, and skinfolds) were performed by trained nurses according to standard procedures, using an electronic scale (Seca 753E; Seca Weighing and Measuring System, ± 100 g), a digital stadiometer (Harpenden, Crymych, UK, ± 0.1 cm), and caliper (Holtain Ltd, Crosswell, Dyfed, UK,  ± 0.1 mm). The participants were thereby dressed in underwear and barefoot.

Body mass index [BMI (kg/m^2^)] was calculated as the body weight (kg) divided by the square of the body height (m^2^). BMI-SDS was calculated based on the German reference percentiles for children and adolescents [17]. Fat mass as well as fat-free mass were estimated from the sum of the subscapular and triceps skinfolds using Slaughter’s equations [18].

The corresponding indices fat mass index (FMI) and fat-free mass index (FFMI) were calculated by dividing the according values by the square of the body height (m^2^).

### Assessment of potential confounding factors

Potential confounding factors were selected according to the known predictors for BMI and body composition [19].

In addition to sex (boy/girl), early life factors such as gestational duration (weeks), maternal weight gain during pregnancy (kg), birth weight (g), and postnatal factors such as full breastfeeding duration (< 4 months, 4–6 months, > 6 months), were considered as possible confounders.

Based on the anthropometric data as well as social indicators of the parents, maternal overweight status (maternal normal weight: BMI < 25 kg/m^2^; maternal overweight BMI: ≥ 25 to < 30 kg/m^2^; maternal obesity BMI: > 30 kg/m^2^) and socio-economic factors such as high maternal educational status (≥ 12 school years yes/no), and maternal employment (yes/no) were derived. Individual sleep duration as well as data on smoking in the household were collected with a standardized questionnaire.

Missing values (pregnancy duration *n* = 22 participants, pregnancy weight gain *n* = 55, birth weight *n* = 10, maternal overweight *n* = 22, maternal educational status *n* = 3, and smoker in household *n* = 201) were replaced by the respective median of the total sample.

### Statistical analysis

All statistical analyses were carried out using SAS^®^ procedures (version 9.40; Cary, NC, USA). The significance level was set at *p* < 0.05.

Descriptive data are shown as median with their interquartile range or frequencies and percentages.

Polynomial mixed-effects regression models (PROC MIXED procedure in SAS), including both fixed and random statements, were used to analyse the association between individual change in dairy intake and concomitant change in body weight status. A repeated statement was considered to account for the lack of independence between repeated measurements from the same individual. Random effects were considered to allow for variation between individuals and families in the baseline (intercept) level of each outcome.

The applied change-on-change model [20] is structured as follows:

- the association between dairy intake at baseline (first examination) and body weight status variables at baseline was examined cross-sectionally (*β*1);
- the association between dairy intake at baseline and change in body weight status over time was analysed (0 for first assessment) (*β*2);
- the association between change in dairy intake and concomitant change in body weight status variables was investigated (*β*3).

All basic models included dairy intake at baseline in 100 g/1000 kcal (*β*1), the interaction of dairy intake at baseline with time (*β*2), the change in dairy intake, defined as the difference between intake in the respective study year and intake at baseline (*β*3), time in years (each defined as 0 for the first individual measurement), and age in years as independent variables.

For adjusted models, potential confounders were included if they had a significant and independent association with the outcome variable (*p* < 0.05), if the regression coefficients in the basic models were modified by ≥ 10% or if they led to an improvement in AIC (Akaike information criterion) of more than two points. Regarding the comparability between models, individual models were calculated in advance for each outcome to identify all significant covariates. Based on these results, a standard adjusted model was built in which all relevant covariates were defined. Hence, the final adjusted models included sex, birth weight, maternal overweight, pregnancy weight gain, and breastfeeding duration.

The associations between dairy intake: TD, LD, SD, NFD, FD, LFD, HFD, HSD, and LSD, respectively, and the respective outcome variables BMI-SDS, FMI, and FFMI were calculated in separate models.

Finally, to adjust for multiple testing, the Benjamini–Hochberg false-discovery-rate-method was used (PROC MULTTEST procedure in SAS).

As no significant interactions of sex and age were observed, no stratification by sex was performed.

The following sensitivity analyses were performed. First, final models were additionally adjusted for energy intake, to prove whether the calculation in 100 g/1000 kcal was sufficiently energy-adjusted [21].

Second, to reduce potential bias, we excluded underreported records. Dietary records were considered as “underreported” if the total energy intake was insufficient in relation to the estimated basal metabolic rate (BMR) according to age- and sex-specific equations by Schofield [22]. Underreported records were identified using the paediatric cut-offs by Sichert-Hellert et al. [23]. This calculation resulted in a total of 794 (7.9%) underreported records.

## Results

The sample of the present evaluation includes all available complete dietary records (*n* = 9999) of *n* = 1126 participants (boys *n* = 572, 50.8%) aged 3.5–18.5 years collected between August 1985 and June 2019. Sample characteristics at first and last assessment are shown in Table 2. The median follow-up time was 9 years. The overweight status and maternal characteristics of the participants reflect the high socio-economic status of the DONALD study participants.

**Table 2:** Baseline and last follow-up characteristics of *n* = 1126 participants (age 3.5–18.5 years, *N* = 9999 3-day dietary records) of the DONALD study between 1985 and 2019

	Baseline	Last measurement	Total
*n*^participants^	1126	1126	1126
Male	572 (50.8)	572 (50.8)	572 (50.8)
*n*^3-day-dietary-records^	1126	1126	9999
Age (years)	4.1 (4.0; 4.3)	14.3 (10.0; 18.1)	9.2 (6.1; 13.1)
Follow-up period/participant (years)			9 (4; 14)
Anthroprometics			
BMI-SDS (kg/m^2^)	0.09 (-0.43; 0.64)	0.07 (-0.63; 0.76)	0.02 (-0.60; 0.66)
FMI (kg/m^2^)	2.38 (2.01; 2.88)	3.58 (2.28; 5.44)	2.72 (2.05; 4.08)
FFMI (kg/m^2^)	13.18 (12.64; 13.82)	15.12 (13.76; 16.78)	13.96 (13.10; 15.24)
Dairy intake			
Total energy intake (kcal/day)	1239.8 (1086.5; 1422.1)	1816.5 (1500.3; 2206.7)	1635.9 (1356.0; 1967.8)
Total dairy (g/1000 kcal)	221.8 (146.2; 300.9)	148.7 (90.2; 217.9)	177.4 (110.8; 254.8)
Liquid dairy (g/1000 kcal)	128.6 (54.2; 207.4)	54.3 (0; 123.5)	82.3 (16.5; 152.8)
Solid dairy (g/1000 kcal)	74.4 (40.9; 117.0)	72.7 (34.9; 120.6)	79.2 (44.1; 123.4)
Fermented dairy (g/1000 kcal)	40.5 (14.3; 73.7)	36.6 (12.5; 67.1)	38.3 (14.7; 70.0)
Not fermented dairy (g/1000 kcal)	162.6 (93.4; 241.2)	100.7 (44.4; 167.6)	127.7 (61.4; 200.9)
Low-fat dairy (g/1000 kcal)	28.4 (2.9; 102.3)	30.4 (0.9; 94.2)	31.1 (2.2; 105.3)
High-fat dairy (g/1000 kcal)	139.5 (61.8; 233.7)	72.7 (33.4; 149.2)	97.5 (40.4; 180.5)
Low-sugar dairy (g/1000 kcal)	139.2 (60.1; 224.9)	99.3 (46.9; 167.9)	115.3 (54.6; 190.3)
High-sugar dairy (g/1000 kcal)	56.4 (25.1; 105.3)	31.0 (9.9; 62.7)	42.2 (15.9; 78.6)
Underreporting	33 (2.9)	162 (14.4)	794 (7.9)
Maternal characteristics			
Maternal overweight^a^			3521 (36)
High educational status^b^			6407 (64)
Employment			6086 (61)
Early life factors			
Pregnancy duration (weeks)^c^			40.0 (39.0; 41.0)
Pregnancy weight gain (kg)^d^			13.0 (10.0; 15.0)
Birth weight (g)^e^			3450 (3130; 3780)
Breastfeeding duration^f^			0 = 2572/1 = 504/2 = 6923
Smokers in household^g^			0 = 6186/1 = 1860

Values are medians (25th, 75th percentile) or frequencies (%)

^a^BMI > 25 kg/m^2^ 22 missings

^b^> 12 year school education 3 missings

^c^22 missings

^d^55 missings

^e^10 missings

^f^0 =  < 4 months/1 = 4–6 months/2 =  > 6 months

^g^0 = no, 1 = yes, 201 missings

The results of the change-on-change analyses are shown in Table 3.

**Table 3:** Regression coefficients of change-on-change analyses among *n* = 1126 DONALD participants (*N* = 9999 records, collected between 1985 and 2019)

	BMI-SDS						FMI						FFMI					
	Baseline intake in 100 g/1000 kcal		Baseline intake in 100 g/1000 kcal*time		Change of intake in 100 g/1000 kcal		Baseline intake in 100 g/1000 kcal		Baseline intake in 100 g/1000 kcal*time		Change of intake in 100 g/1000 kcal		Baseline intake in 100 g/1000 kcal		Baseline intake in 100 g/1000 kcal*time		Change of intake in 100 g/1000 kcal	
	*β*	*p* value	*β*	*p* value	*β*	*p* value	*β*	*p* value	*β*	*p* value	*β*	*p* value	*β*	*p* value	*β*	*p* value	*β*	*p* value
Total dairy																		
Model 1	0.0301	(0.2487)	− 0.0010	(0.7612)	0.0092	(0.0388)	− 0.0010	(0.9552)	0.0115	(0.1251)	0.0216	(0.0176)	0.0454	(0.0880)	− 0.0110	(0.0049)	0.0159	(0.0388)
Model 2	0.0344	(0.1474)	− 0.0009	(0.7688)	0.0092	**(0.0371)**	0.0044	(0.8895)	0.0116	(0.1166)	0.0220	**(0.0162)**	0.0516	**(0.0342)**	− 0.0110	**(0.0052)**	0.0156	**(0.0417)**
Liquid dairy																		
Model 1	0.0163	(0.6076)	0.0013	(0.7077)	0.0139	(0.0054)	0.0019	(0.9411)	0.0248	(0.0014)	0.0252	(0.0145)	0.0311	(0.2923)	− 0.0110	(0.0062)	0.0242	(0.0049)
Model 2	0.0228	(0.3904)	0.0013	(0.6978)	0.0139	**(0.0052)**	0.0071	(0.8119)	0.0249	**(0.0014)**	0.0258	**(0.0125)**	0.0410	(0.1160)	− 0.0110	**(0.0074)**	0.0239	**(0.0052)**
Solid dairy																		
Model 1	0.0608	(0.2469)	− 0.0080	(0.1251)	− 0.0030	(0.6933)	− 0.0100	(0.8483)	− 0.0460	(0.0014)	0.0036	(0.8398)	0.0672	(0.2364)	− 0.0006	(0.9411)	− 0.0080	(0.5726)
Model 2	0.0546	(0.2673)	− 0.0080	(0.1201)	− 0.0040	(0.6978)	− 0.0080	(0.8895)	− 0.0460	**(0.0014)**	0.0036	(0.8621)	0.0554	(0.2920)	− 0.0009	(0.9285)	− 0.0090	(0.5221)
Fermented dairy																		
Model 1	0.1618	(0.0054)	− 0.0007	(0.9370)	0.0050	(0.6778)	0.0653	(0.2752)	− 0.0170	(0.4109)	0.0190	(0.3415)	0.2093	(0.0014)	− 0.0130	(0.2259)	0.0126	(0.4969)
Model 2	0.1397	**(0.0133)**	− 0.0004	(0.9571)	0.0053	(0.6366)	0.0528	(0.3779)	− 0.0170	(0.3989)	0.0194	(0.3452)	0.1899	**(0.0014)**	− 0.0130	(0.2074)	0.0133	(0.4370)
Not fermented dairy																		
Model 1	0.0034	(0.9227)	− 0.0008	(0.7832)	0.0092	(0.0540)	− 0.0120	(0.6873)	0.0142	(0.0483)	0.0194	(0.0483)	0.0110	(0.7523)	− 0.0090	(0.0211)	0.0147	(0.0824)
Model 2	0.0119	(0.6978)	− 0.0008	(0.7970)	0.0092	(0.0550)	− 0.0040	(0.8895)	0.0145	**(0.0417)**	0.0198	**(0.0417)**	0.0210	(0.4370)	− 0.0090	**(0.0226)**	0.0142	(0.0928)
Low-fat dairy																		
Model 1	0.0641	(0.0211)	− 0.0030	(0.4109)	0.0056	(0.3407)	0.0066	(0.8398)	0.0345	(0.0014)	0.0194	(0.0886)	0.0981	(0.0014)	− 0.0220	(0.0014)	0.0007	(0.9411)
Model 2	0.0593	**(0.0275)**	− 0.0030	(0.4348)	0.0055	(0.3491)	− 0.0010	(0.9705)	0.0348	**(0.0014)**	0.0196	(0.0859)	0.1016	**(0.0014)**	− 0.0220	**(0.0014)**	0.0002	(0.9753)
High-fat dairy																		
Model 1	− 0.0170	(0.5726)	0.0012	(0.7268)	0.0066	(0.2259)	− 0.0080	(0.7724)	− 0.0180	(0.0157)	0.0102	(0.3415)	− 0.0280	(0.3407)	0.0065	(0.1251)	0.0176	(0.0368)
Model 2	− 0.0090	(0.7688)	0.0011	(0.7466)	0.0067	(0.2006)	0.0035	(0.9024)	− 0.0180	**(0.0157)**	0.0105	(0.3446)	− 0.0230	(0.3963)	0.0062	(0.1474)	0.0177	**(0.0342)**
Low-sugar dairy																		
Model 1	0.0160	(0.6076)	− 0.0040	(0.1641)	0.0133	(0.0041)	− 0.0080	(0.7724)	0.0025	(0.7724)	0.0196	(0.0368)	0.0319	(0.2752)	− 0.0120	(0.0035)	0.0187	(0.0167)
Model 2	0.0198	(0.4370)	− 0.0040	(0.1530)	0.0132	**(0.0041)**	− 0.0040	(0.9024)	0.0028	(0.7688)	0.0200	**(0.0316)**	0.0357	(0.1712)	− 0.0120	**(0.0035)**	0.0183	**(0.0189)**
High-sugar dairy																		
Model 1	0.0449	(0.3407)	0.0081	(0.0634)	− 0.0090	(0.2487)	0.0231	(0.6569)	0.0312	(0.0167)	0.0063	(0.7415)	0.0474	(0.3407)	0.0011	(0.9096)	− 0.0050	(0.7415)
Model 2	0.0470	(0.2761)	0.0083	(0.0556)	− 0.0090	(0.2565)	0.0256	(0.5754)	0.0310	**(0.0172)**	0.0063	(0.7466)	0.0555	(0.2181)	0.0009	(0.9278)	− 0.0050	(0.7616)

All model 1 were adjusted for age and time

All model 2 were adjusted for sex, birth weight, maternal overweight, weight gain during pregnancy, and breastfeeding

*p* values were additionally adjusted using the false discovery testing method

Overall, an increase in TD intake of 100 g/1000 kcal was only marginally but significantly associated with an increase in BMI-SDS (*β* = 0.01; *p* = 0.04), an increase in FMI (*β* = 0.02; *p* = 0.02), as well as an increase in FFMI (*β* = 0.02; *p* = 0.04). Related to the median baseline measures in body composition, the observed association would correspond to a 10.2% increase in BMI-SDS, a 0.9% increase in FMI, and a 0.1% increase in FFMI.

Dairy type analyses for LD and LSD showed similar results. An increase in LD intake yielded an increase in BMI-SDS (*β* = 0.01; *p* = 0.01), in FMI (*β* = 0.03; *p* = 0.01), and in FFMI (*β* = 0.02; *p* = 0.01). This corresponds to a 15.4% increase in BMI-SDS, a 1.1% increase in FMI, and a 0.2% increase in FFMI compared to baseline.

Also an increase in LSD intake resulted in an increase in BMI-SDS (*β* = 0.01; *p* = 0.004), in FMI (*β* = 0.02; *p* = 0.03), and in FFMI (*β* = 0.02; *p* = 0.02) per 100 g/1000 kcal. As expressed in percentage, there was a 14.7% increase in BMI-SDS, a 0.8% increase in FMI, and a 0.1% increase in FFMI compared to baseline.

However, the processing method (fermentation) as well as the fat content showed no association with body composition in our analyses after conducting sensitivity analyses with additional adjustment of energy intake as well as sensitivity analyses excluding the underreported records.

For other dairy types, the sensitivity analyses showed similar results to the main analyses [supplementary table (S1)].

## Discussion

The present study investigated associations between changes in TD and dairy types and concomitant changes in BMI-SDS, FMI, and FFMI among children and adolescents. To the best of our knowledge, comparable analyses were not examined earlier.

Our analyses showed that TD intake had a significant positive association with BMI, while changing both fat mass and fat-free mass. However, in percentage, we found only a notable increase in BMI-SDS of 10.2% as compared to baseline. In contrast, the percentages for FMI (0.9%) and FFMI (0.1%) were negligible.

Partially similar results were provided by a meta-analysis of randomized controlled trials in children and adolescents by Kang et al. [5], who reported a positive association between dairy intake and body weight and fat-free mass. In contrast a meta-analysis in children by Lu et al. [4] revealed no association with BMI but an inverse association with the risk of overweight or obesity at an average 3-year follow-up. However, Kang et al. and Lu et al. summarized different studies on different types of dairy on dairy intake in general, so the results are difficult to compare.

Because of differences in compositions and structures of dairy types, it was reasonable to assume that intakes of specific types of dairy may have different health effects [24]. For this reason, we additionally analysed the association between the intake of different dairy types and body composition.

In our analyses, we found a significant positive association between LD intake and BMI-SDS, FMI, and FFMI, although again only the percentage change in BMI-SDS (15.4%) related to baseline value was meaningful. Wang et al. [8] also analysed the association of milk intake on the risk of obesity in children and adults, which was defined as increased percentage body fat or increased BMI. A higher milk intake led to a lower risk of obesity [8]. However, Wang et al. [8] summarized cross-sectional studies and only considered plain milk but not other liquid-dairy products, such as cocoa or dairy drinks. Also, an umbrella review by Zhang et al. [25] in children and adults showed that milk intake was inversely associated with obesity.

Hartwig et al. [26] investigated the association of milk consumption with obesity using genetically defined lactase persistence from the Pelotas birth cohort and combined the results with the results of a meta-analysis on the association of milk consumption with obesity. He found a positive association of lactase persistence with BMI and overweight and obesity. For further analysis of the effects of milk intake on body composition, the information of lactase persistence in subjects could therefore be useful.

Considering the fat content, the studies included in the systematic review by O’Sullivan et al. [11] consistently reported that full-fat dairy products were not associated with increased weight gain or obesity in children. Some of these studies even show positive associations of overweight and obesity with consumption of reduced-fat dairy products and inverse associations with consumption of full-fat dairy products. In our analyses, neither the intake of low-fat dairy nor the intake of high-fat dairy showed an association with body composition variables. In recent years, a possible link between vitamin D status and obesity has been discussed [27]. In Germany, dairy is not fortified with vitamin D. The naturally higher vitamin D content in high-fat dairy or an interaction of vitamin D with other ingredients such as calcium [28] could possibly compensate the higher fat intake. However, further research is needed to establish this possible link and to investigate whether there is a similar association in countries where dairy is vitamin D fortified.

In terms of sugar content in dairy products, our results showed a positive association between change in LSD intake and change in BMI, FMI, and FFMI, respectively. Again, only the percent change related to baseline value in BMI-SDS (14.7%) showed meaningful values. It should be noted that in the present investigation, only industrially sweetened dairy has been taken into account, whereas sugar or other sweeteners added to dairy products at home were not considered. Therefore, the results may differ if household added sugar in dairy is considered. However, in our analyses, a higher HSD intake did not led to a change in BMI and body composition. A narrative review by Fayet-Moore [29] also concluded that there is no association between flavoured milk consumption and BMI, prevalence of obesity, or prospective change in BMI in normal-weight children. Conflicting effects were observed in overweight children. Previous trend analyses with DONALD participants showed a fluctuating trend in the intake of HSD over time [10, 30]. Nevertheless, the intake of low-sugar varieties should be recommended, as the intake of free sugars in Germany exceeds the limit of 10% of energy intake [30, 31].

To our knowledge, there is no analysis of the relationship between fermented dairy intake and obesity in children and adolescents. A meta-analyses in adults by Schwingshackl et al. [12] found an inverse relationship between yoghurt and cheese intake and body weight. Also other studies among adults showed a beneficial association between yogurt intake and body composition [32, 33]. In our analyses, no associations were observed for changes in either NFD or FD intake with body composition changes in children and adolescents. The different results could be due to the fact that we have combined fermented dairy, e.g., yoghurt and cheese, into one group rather than looking at these fermented dairy products separately, although these groups are different in their matrices of nutrients and therefore could have different effects on body weight. However, the overall intake of fermented dairy in our collective was too low to subdivide it. A meta-analysis of randomized controlled trials in adults by Borgeraas et al. [34] resumed an administration of probiotics resulted in a significantly larger reduction in body weight, BMI, and fat percentage, compared with placebo, but the effect sizes were small. It would be worth investigating whether the administration of probiotics has an effect on body composition in children and adolescents.

Overall, although dairy intake has a significant association with body composition variables, this appears to be small. A meta-analysis of clinical trials in adults by Onvani et al. [35] showed that consumption of dairy products significantly increases the feeling of satiety, which could explain the low influence on body composition. Overall, the nutritional and satiety benefits of dairy intake in children and adolescents outweigh the effects on body composition.

Some strengths and limitations of the DONALD study and the present investigation need to be discussed: The major strength of the DONALD study is its longitudinal design with closed-meshed measurements, which allows analyses of the association of dairy intake on body composition with a large sample size using a large number of 3-day weighted dietary records. The continuously updated in-house nutrient database LEBTAB allows the consideration of different types of dairy according to composition and processing methods [16]. A limitation of the present study is the overrepresentation of families with a high socio-economic status in the collective of the DONALD study, which limits the generalisability of our results [15]. However, in our previously conducted trend analysis with the same sample, the dairy intake data were comparable to the results of the German National Nutrition Survey (NVS II) [10, 36]. Furthermore, we cannot rule out the possibility of underreporting. Underreported records were not generally excluded from the main analyses, as this method only identifies underreported energy intake, but not selective underreporting of single foods [37]. Furthermore, participants with high-energy requirements, who may have been underreported, could not be identified [38]. However, our sensitivity analyses showed similar results after excluding underreported data sets (supplementary table S1). Physical activity, assessed via questionnaire [15] since 2004, could not be included due to insufficient data availability (missing *n* = 756 participants).

## Conclusion

Our analyses of dairy intake in children and adolescents with a mean follow-up of 9 years from 1985 to 2019 showed small but significant associations of TD, LD, and LSD intake and BMI as well as fat mass and lean mass. However, the observed changes were too small to expect biologically or physiologically significant effects. The processing method as well as the fat content of dairy showed no association with body composition in our analyses. Overall, our results show that measures to promote dairy consumption in childhood are to be welcomed, as no negative effects on body composition are to be expected, while the intakes of important nutrients for growth and satiety are ensured. The type of dairy does not seem to be important.

## Supplementary Information

Below is the link to the electronic supplementary material.

Supplementary file1 (XLSX 20 KB)
